# The role of internet-based digital tools in reducing social isolation and addressing support needs among informal caregivers: a scoping review

**DOI:** 10.1186/s12889-019-7837-3

**Published:** 2019-11-09

**Authors:** Kristine Newman, Angel He Wang, Arthur Ze Yu Wang, Dalia Hanna

**Affiliations:** Ryerson University, 350 Victoria Street, Toronto, Ontario M5B 2K3 Canada

**Keywords:** Caregivers, Digital tools, Internet, Social isolation, Informal caregivers, Digital technology

## Abstract

**Background:**

In Canada, 8.1 million people informally provide care without payment, primarily to family members; 6.1 million of them are employed at a full-time or part-time job. Digital technologies, such as internet-based tools, can provide informal caregivers’ access to information and support. This scoping review aimed to explore the role of internet-based digital tools in reducing social isolation and addressing support needs among informal caregivers.

**Methods:**

A systematic search for relevant peer-reviewed literature was conducted of four electronic databases, guided by Arksey and O’Malley’s framework. An extensive search for relevant grey literature was also conducted.

**Results:**

The screening process yielded twenty-three papers. The following themes were generated from the reviewed studies: searching for and receiving support; gaining a sense of social inclusion and belonging; and benefits and challenges of web-based support. The studies noted that, to connect with peers and obtain social support, informal caregivers often turn to online platforms. By engaging with peers in online communities, these caregivers reported regaining a sense of social inclusion and belonging.

**Conclusions:**

The findings suggest that internet-based digital tools can be a cost-effective and convenient way to develop programs that help unpaid caregivers form communities, gain support, and access resources. Service providers can leverage digital tools to deliver support to caregivers within online communities.

## Background

A growing number of unpaid caregivers of all ages use the internet as a primary source of information, although there are concerns about the reliability of web-based information. Unpaid caregivers work with vulnerable people, particularly if the people they care for have complex needs. Although such people can also be identified as carers or care partners, for this scoping review, we will use the term ‘informal caregivers.’ The Change Foundation defines informal caregivers as “the people – family, friends, neighbours – who provide critical and ongoing personal, social, psychological and physical support, assistance and care, without pay, for loved ones in need of support due to frailty, illness, degenerative disease, physical/cognitive/mental disability, or end of life circumstances.” [1] Many caregivers do not immediately identify themselves as such; instead, they see their role as parent, sibling, child, spouse, friend, or neighbour. Nonetheless, the demands placed on them significantly extend beyond the common expectations of these roles [2]. Most caregivers find their roles both rewarding and challenging, depending on the day or situation, and long-term caregiving often leads to caregiver burnout [2].

The National Institute on Ageing recommends that Canada’s provincial, territorial, and federal governments formally recognize caregivers with a common definition that acknowledges their role and lays the foundation for the development of formal support [3]. On April 3, 2018, the Premier of Canada’s largest province (Ontario), Kathleen Wynne, announced the Ministry of Health and Long-Term Care’s intention to provide $6.5 million to create a new, not-for-profit entity to serve the province: the Ontario Caregiver Organization [4]. The new entity will engage with informal caregivers and other organizations to understand their needs and develop caregiver-centred supports accordingly [5].

Informal caregivers remain largely invisible, and their role is inadequately recognized [3]. In Canada, 8.1 million people are caregivers, and 6.1 million of them (75% of caregivers; 35% of the total Canadian labour force) must balance employment and caregiving responsibilities [6]. In 2017, the estimated cost of caregiving in Canada was $33 billion [7]. One million people (31% of caregivers) reported that they had no choice in taking on their caregiving responsibilities [8].

In Ontario, an estimated 3.3 million people (29% of the provincial population) are informal caregivers, and most (84%) care for a family member [2]. These caregivers comprise an equal proportion of women (53%) and men (47%) [2]. Ontario informal caregivers vary in age: 23% are between the ages of 45 and 54; 19% are 55 to 64; 17% are 15 to 24; and 11% are 65 or over. There are no data on caregivers under the age of 15 [8]. More than a quarter of Ontario residents were born outside the country. This diversity is reflected in the Ontario caregiver population, considering that 21% of caregivers reported their primary language to be other than English or French [8].

Ontario’s informal caregivers spend an average of 11 h a week providing care [2]. The care comprises emotional support and companionship (90% of caregivers), transportation (79%), indoor domestics tasks (meal preparation, house cleaning, and laundry) (57%), outdoor tasks (home and property maintenance) (53%), medical treatments (e.g., tube feeding) (29%), scheduling and coordinating appointments (31%), and personal care (e.g., bathing, toileting, eating, mobility and personal hygiene) (22%) [8].

Digital technologies, such as internet-based tools, can support informal caregivers’ access to information and connect them. The ability for caregivers to connect is essential, and digital tools can also link them to other caregivers who face similar issues in supporting their families. Digital tools can help caregivers raise awareness of these issues, foster deeper understanding of the issues faced by those they care for, and create a collective community for the public. Although informal caregivers are significantly more likely to seek medical information online compared with the general public, they are significantly less likely to use mobile applications for this purpose [9]. However, informal caregivers tend to commonly use mobile apps to access social support through social media platforms such as Facebook [10].

Social isolation is “a state in which the individual lacks a sense of belonging socially, lacks engagement with others, has a minimal number of social contacts and they are deficient in fulfilling and quality relationships”. ( [11], (p1346)) The social isolation of informal caregivers has serious consequences, because they are exposed to the chronic suffering of loved ones, the impact of injury, the dying process, and the aftermath of death. As the informal-caregiver population increases, there is a need for greater awareness of how digital tools can support their valuable work and enhance their mental well-being.

The opposite of social isolation, social inclusion, “refers to three central characteristics at the interface of individuals and their environments: (a) social integration; (b) social support; and (c) access to resources”. ( [12], (p113)) The first central characteristic, “Social integration [,] reflects the extent to which individuals are embedded within a network of meaningful social bonds and societal structures, as evidenced by the size, density and intensity of social relationships and contact” ( [12], (p113)).

The second central characteristic of social inclusion, “Social support [,] refers to the extent to which those social bonds enable network members to obtain help when they need it.” ( [12], (p113)). There are four main types of social support: emotional, appraisal, informational, and instrumental [12]. Emotional support pertains to the sharing of life experiences and providing empathy, trust, and caring [13]. Appraisal support relates to providing information that is useful for self-evaluation, such as constructive feedback, affirmation, and social comparison [13]. Informational support involves providing guidance, advice, and information that individuals can use to address their challenges [13]. Lastly, instrumental support comprises tangible aid and services that can directly help the person in need [13].

The third central characteristic of social inclusion, resource access, reflects the perception that “social relationships serve a potential instrumental function in promoting the wellbeing and fulfilment of network members.” ([12)] (p 113)).

### Aims

Our scoping review aimed to explore the nature and extent of the role that internet-based digital tools play in reducing informal caregivers’ social isolation and addressing their support needs. In particular, we sought to answer the following questions:

1. Which internet-based digital tools do informal caregivers use for social support?
2. How do internet-based digital tools reduce social isolation among and address the support needs of informal caregivers?
3. How do informal caregivers experience internet-based digital tools for social support?
4. Do informal caregivers have any unmet needs that could be met by internet-based digital tools for social support?

## Methods

Our scoping review was guided by Arksey and O’Malley’s methodology [14] and included the following steps: (1) identifying the research question; (2) searching for relevant references; (3) selecting references to review; (4) charting the data; and (5) collating, summarizing, and reporting the results. In the second step, a medical research librarian searched the following databases: Medline, EMBASE, PsycINFO, and CINAHL-EBSCO. The search combined terms for informal caregivers and online social communities. All database searches were updated December 10, 2017. For the detailed search strategies, see Additional file 1.

In addition to the database searches, an extensive grey literature search was conducted. This included a search targeting relevant government and research websites as well as those identified in the document “Organizations Advocating for and Supporting Family Caregivers.” [15] A series of broader grey-literature searches were also undertaken, using the Google Advanced search engine. These searches combined the terms for informal caregivers and online social communities used in the database search, and the first 150 results were scanned for relevance. Finally, after studies for chosen for review, their reference lists were examined for relevant studies not found in the database and grey-literature searches (see Additional file 1).

In the third step, after the searches were complete, the references’ titles and abstracts were screened for relevance independently by two reviewers (AHW and AZYW), according to the following inclusion criteria: (1) full text available through the Ryerson University library database; (2) written in English; (3) empirical study or systematic review; and (4) explicitly explored the use of internet-based digital technology tools by informal caregivers to reduce social isolation and to address their support needs. References were excluded if (1) their samples included formal caregivers (e.g., doctors, nurses, personal support workers); (2) were not published between January 1, 2005 and November 15, 2017; (3) explicitly explored the use of digital technology tools by informal caregivers that were not internet-based; and (4) evaluated only usability and acceptability of the internet-based digital too (i.e., no focus on social supports only using the technology). The two reviewers and KN, the third reviewer, met after reviewing a number of references to ensure they consistently interpreted the inclusion and exclusion criteria. When there were disagreements, all three reviewers came to consensus. When titles and abstracts passed the screening, the full article was obtained and then screened again by AHW and AZYW, using the same inclusion criteria. Again, disagreements were resolved through discussion among all three reviewers until consensus was reached.

In the fourth step, details were charted of how references that passed both screenings met the inclusion criteria and their context. A narrative synthesis of the findings was completed by AHW, ZYW and KN to explore digital inclusion, the social exchanges among informal caregivers facilitated by internet-based tools.

## Results

**Fig. 1:**
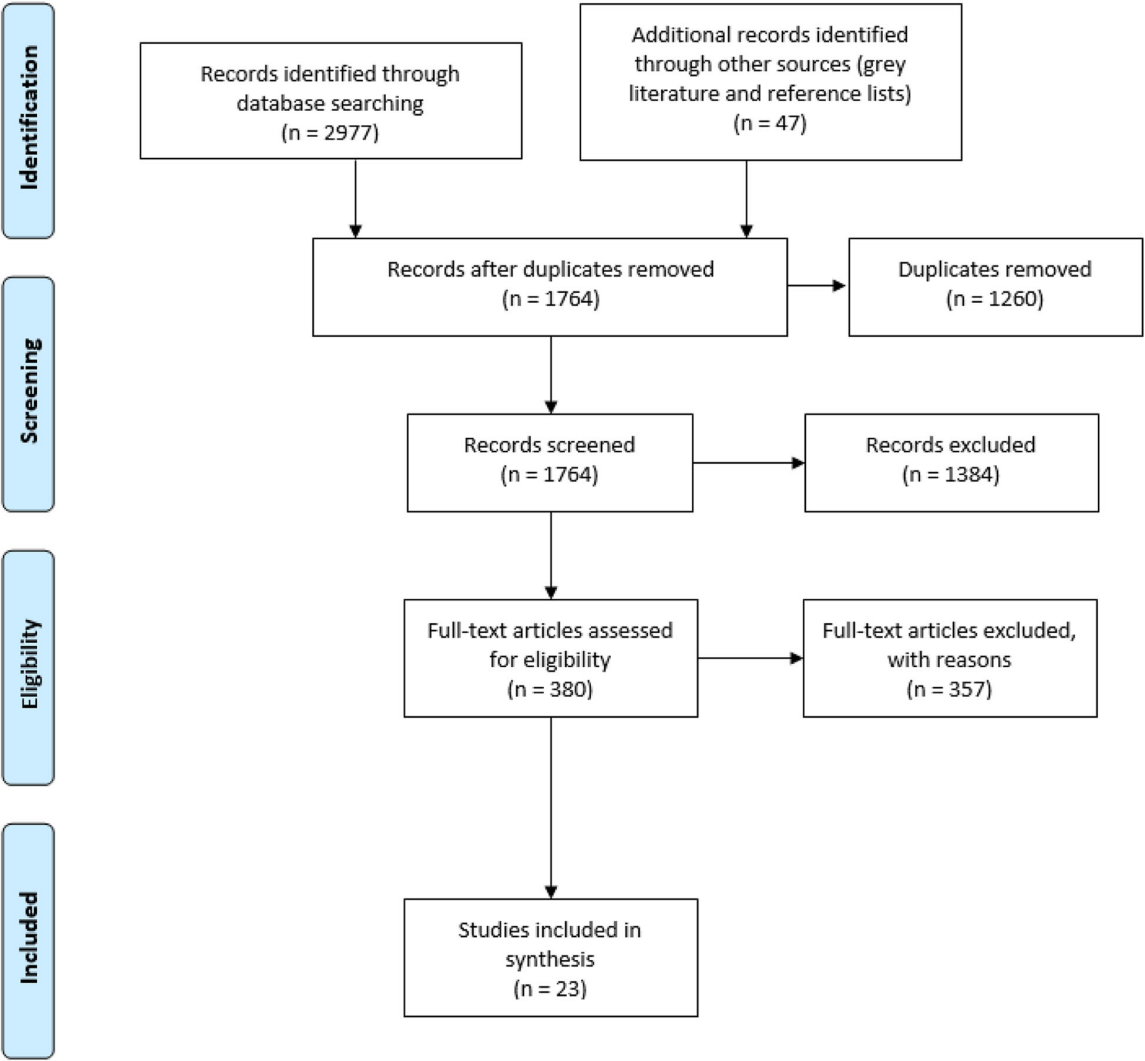
Search strategy and its results, Format of figure based on Moher et al. [16]

Searching online databases for scholarly, peer-reviewed literature and gleaning the reference lists of references selected for review identified 2977 articles. An additional 47 references were found through an Advanced Google search of grey literature. In total, 3024 articles were identified. After removing duplicates, the titles and abstracts of 1764 of these articles were screened using the inclusion and exclusion criteria. This screening resulted in 380 articles being eligible for full-text screening. A second screening of the full-text articles resulted in 23 being selected for the scoping review. See Fig. 1 for the flowchart of the study screening and selection process.

### Characteristics of reviewed articles

Table 1 summarizes characteristics of the reviewed articles. Study sample sizes ranged from six to 860 participants. Study participants ranged from 18 to 86 years of age. Most of the studies that collected information about participants’ gender and ethnicity included more women than men and reported that participants were mostly of European descent. Of the 23 studies, 11 were conducted in the United States. Other countries where studies were conducted include Canada (3), Sweden (3), United Kingdom (2), and one each in Italy, Malaysia, Netherlands, and Poland.

**Table 1:** Characteristics of reviewed articles

Authors [reference number]	Purpose of study	Sample size	Target population	Age of caregivers (years)	Method	Gender and ethnicity of caregivers	Residing country of first author
Anderson et al. [17]	To explore how family caregivers of persons living with dementia use the social media platform of the blog as a part of the individual caregiving experience.	9	Caregivers of persons living with dementia	N/A	Qualitative: Analyzed samples of blog content using content analysis supplemented by thematic analysis	Gender: 8 women 1 man Ethnicity: N/A	United States
Barbabella et al. [18]	To verify the impact of a web-based psychosocial intervention on caregivers.	94, in 20 focus groups	Caregivers of older adults	51–69	Pretest-posttest design with mixed methods: Questionnaires and focus groups	Gender: 57 women 37 men Ethnicity: N/A	Italy
Bateman et al. [19]	To determine (1) the feasibility of innovating peer support group work delivered through social media with friendsourcing; (2) whether the intervention provides an acceptable method for AD caregivers to obtain support; and (3) whether caregiver outcomes were affected by the intervention.	6	Caregivers of persons living with Alzheimer’s disease	34–74	Pretest-posttest design with mixed methods: Surveys and semi-structured interviews	Gender: 3 women 3 men Ethnicity: 4 White 1 African American 1 Asian American	United States
Blusi, Asplund, Jong [20]	To illuminate the meaning of ICT-based caregiver support as experienced by older family carers living in vast rural areas, caring for a spouse at home.	31	Caregivers in rural areas	65–85	Qualitative, descriptive design: Semi-structured interviews	Gender: 23 women 8 men Ethnicity: N/A	Sweden
Blusi, Kristiansen, Jong [21]	To explore how internet-based caregiver support may influence the experience of isolation among older spousal caregivers in rural areas.	31	Older spousal caregivers in rural areas	65–85	Qualitative: Interviews post-intervention	Gender: 23 women 8 men Ethnicity: N/A	Sweden
Dam et al. [22]	To describe (1) the development of an online social support intervention titled Inlife; and (2) the evaluation of the feasibility of this intervention and the measurements to assess its effectiveness.	25	Caregivers of persons living with dementia	Mean = 55.9 (SD = 13.9)	Quantitative: Online self-report measures at baseline and at four follow-up time points	Gender: 12 women 13 men Ethnicity: N/A	Nether-lands
Darcy, Brunsden, Hill [23]	To explore the use of an online mental health discussion board by informal caregivers.	82	Caregivers of persons living with mental health issues	N/A	Qualitative: Interpretative phenomeno-logical analysis of 487 postings	Gender: N/A Ethnicity: N/A	United Kingdom
Diefenbeck, Klemm, & Hayes [24]	To examine content themes emerging from an unstructured, asynchronous online peer support group for family caregivers of persons living with chronic illness.	16	Caregivers of family members living with chronic disease	38–70	Qualitative, descriptive, exploratory design: Qualitative content analysis of the written interactions of the support group members	Gender: 16 women Ethnicity: 16 White	United States
Fox & Brenner [25]	To provide an overview of the demographics of caregivers in USA and how they use the internet.	860	Caregivers of family members	N/A	Quantitative: Surveys	Gender: 478 women 392 men Ethnicity: 626 White 104 Black 96 Hispanic	United States
Gage-Bouchard et al. [26]	To examine the nature of support exchanges between parents of pediatric cancer patients as they happen in real-time, naturally occurring interactions on Facebook.	N/A	Caregivers of persons living with cancer	N/A	Qualitative: Content analysis of 12 months of data from 18 publicly available Facebook pages hosted by parents of children with acute lymphoblastic leukemia	Gender: N/A Ethnicity: N/A	United States
Gage-Bouchard et al. [27]	To examine how cancer caregivers use personal Facebook pages for cancer-related communication.	N/A	Parents of children living with acute lymphoblastic leukemia	N/A	Qualitative: Content analysis of 12 months of data from 18 publicly available Facebook pages hosted by parents of children with acute lymphoblastic leukemia	Gender: N/A Ethnicity: N/A	United States
Hansen, Sheehan, Stephenson [28]	To explore the lived experience of caregivers who interact with their loved ones with a life-limiting illness on an illness blog.	9	Caregivers of ill family members	N/A	Qualitative, phenomenolog-ical approach: Semi-structured interviews	Gender: 5 men 4 women Ethnicity: N/A	United States
Kim [29]	To utilize a uses and gratifications framework to examine how caregivers of children with Down syndrome use social media to access social support.	100	Caregivers of children living with Down Syndrome	18–29 (*n* = 8) 30–39 (*n* = 51) 40–49 (*n* = 38) 50+ (*n* = 3)	Mixed-methods: First phase used a cross-sectional survey research design; second phase used thematic content and feature analysis of social media sites	Gender: N/A Ethnicity: N/A	United States
Kruk [30]	To explore how the situatedness, multiplicity and fragmentation of story forms and functions enable Alzheimer’s caregivers to invoke categories and project multiple troublesome facets of their identities, which then emerge as meaningful amid interactive engagements in an online support group.	N/A	Caregivers of persons living with Alzheimer’s disease	N/A	Qualitative: Conversation analysis and membership categorization analysis of publicly accessible data (15 forum threads) nested within a UK-based online Alzheimer’s support group	Gender: N/A Ethnicity: N/A	Poland
Lichenstein, McDonough, Matura [31]	To gain an understanding of how caregivers of people with pulmonary hypertension are using an online discussion board.	98	Caregivers of persons living with pulmonary hypertension	N/A	Qualitative, descriptive design: Thematic analysis of internet posts on the Pulmonary Hypertension Discussion Board over an 18-month period	Gender: 59 women 18 men Ethnicity: N/A	United States
Marziali & Garcia [32]	To examine the impact on dementia caregivers’ experienced stress and health status of 2 Internet-based intervention programs.	91	Caregivers of persons living with dementia	N/A	Quasi-experimental, mixed-methods design: Questionnaires and qualitative analysis on archived video conferencing group sessions, text-based chat forum exchanges and follow-up interviews	Gender: N/A Ethnicity: N/A	Canada
McKechnie, Barker, Stott [33]	To examine the impact of a UK-based online support forum for caregivers of persons living with dementia.	61	Caregivers of persons living with dementia	22–86	Mixed-methods: Questionnaires and interviews	Gender: 99 women 18 men Ethnicity: 112 White British 4 White other 3 Other	United Kingdom
Roffeei, Abdullah, Basar [34]	To investigate the nature and potential benefits of social support for parents/caregivers of children with Autism Spectrum Disorders using a content-analysis approach to information exchanged via postings and comments within Facebook autism groups.	N/A	Caregivers of persons living with autism	N/A	Qualitative: Deductive content analysis of 3637 messages from two Facebook autism support group pages	Gender: N/A Ethnicity: N/A	Malaysia
Stephen et al. [35]	To report participant and participation characteristics in the pan-Canadian initiative known as CancerChatCanada, and to understand participant perspectives about the quality of communication and professional facilitation, overall satisfaction, and psychosocial benefits and outcomes.	102	Caregivers Cancer patients Cancer survivors	25–79	Qualitative: Interviews	Gender: N/A Ethnicity: N/A	Canada
Stjernswärd, Hansson [36]	To explore participants’ use of a Web-based tool, with focus on the forum, and to assess its potential health and psychosocial benefits.	10	Relatives of persons living with depression	18–68	Mixed-methods: Questionnaires and content analysis of forum posts.	Gender: 9 women 1 man Ethnicity: N/A	Sweden
Sullivan [37]	To gain insight into the “lived” experiences of an online asthma caregivers support group.	31 posters 796 subscrib-ers	Caregivers of persons living with asthma	N/A	Qualitative: Phenomenolog-ical thematic analysis of archived messages of an online asthma caregiver group	Gender: 31 women Ethnicity: N/A	United States
Wasilewski et al. [38]	To explore adult children caregivers’ experiences with online and in-person peer support exchange while caring for an elderly parent.	15	Caregivers who are adult children caregivers of an elderly parent	Mean = 51 (SD = 7.9)	Qualitative, descriptive approach: Semi-structured interviews	Gender: 64 women 7 men Ethnicity: N/A	Canada
Wittenberg-Lyles et al. [39]	To show how bereaved individuals experience loss- and restoration-oriented stressors and how they cope with these distinct types of stressors, and to explore the outcomes of participation in a secret Facebook group for bereavement.	16	Bereaved hospice caregivers	Mean = 48.6 (SD = 16.1)	Mixed-methods: Self-reported measures and content analysis of secret Facebook group online communications	Gender: 11 women 5 men Ethnicity: 15 Caucasian 1 Native American	United States

Note. *N/A* Not available

The target populations of the studies included caregivers of people experiencing or living with Alzheimer’s disease, asthma, autism, cancer, dementia, depression, Down syndrome, acute lymphoblastic leukemia, and pulmonary hypertension. Some studies also had broader target populations, which included caregivers of adult children, family members, and family members with any type of mental health issue. Two studies [20, 21] looked at people who live in rural areas. The types of internet-based resources that were evaluated included private and public discussion forums or blogs (5), peer-to-peer or professionally facilitated support groups (4), and Facebook groups or social media (6) as well as more broadly defined interventions, such as information and communication technology support services and internet-based support services (8).

To evaluate the different online resources, studies employed qualitative (14), mixed (7), and quantitative (2) methods.

### Themes

We identified the following themes among the reviewed studies: (1) searching for and receiving support; (2) gaining a sense of social inclusion and belonging; and (3) benefits and challenges of internet-based support. The themes are discussed in detail below.

#### Searching for and receiving support

Internet-based platforms enabled informal caregivers to access others’ perspectives as well as to gain various forms of support from others. According to Fox and Brenner, caregivers are more likely than other internet users to leverage online social tools related to health [25]. Specifically, caregivers are more likely to read someone else’s personal health story online and to look online for someone with similar health concerns than non-caregivers. This finding corresponds to findings of the other reviewed studies. For example, participants in Lichenstein and colleagues’ study sought support and advice through an online discussion board [31]. They looked to other caregivers for knowledge and advice to assist with their challenges. Posts from caregivers were often heartfelt, as most shared their stories and tried to comfort those in need. When they explored what types of support are exchanged within two Facebook autism-support-group pages, Roffeei and colleagues found that informational and emotional support were most frequently offered and sought by caregivers; they often asked for advice and suggestions and built friendships within the online community [34]. Similarly, when Hansen et al. explored the experiences of family caregivers participating on an illness blog, they found that all participants described wanting to find and accept support from others experiencing similar situations [28].

Sharing is a feature of online support. A study that examined cancer-related Facebook pages found that cancer caregivers used the platform to mobilize emotional, informational, and logistical support as they communicated with others and sought cancer-related advice, resources, and information [26]. Lichenstein et al. elaborated on this finding. They found that caregivers of people with pulmonary hypertension turn to online support groups, seeking other caregivers who share their knowledge and advice and people to listen to their personal stories [31]. Other researchers also found that online support groups offered a forum for sharing personal stories of caregiving experiences with others. These stories satisfy caregivers’ needs for catharsis through venting their emotions as well as exchanging various types of support [24, 35, 37–39].

#### Gaining a sense of social inclusion and belonging

Some caregivers may experience isolation and loneliness as a result of their caregiving role. These feelings often originate from difficulties with leaving the people they care for and from a dearth of friends and family who understand what they are going through. Stjernswärd and Hansson reported that caregivers of people living with depression described their own feelings of loneliness as a “lonesome rollercoaster.” [36] Caregivers discussed how they would feel guilty if they left their care recipients alone to socialize. This guilt resulted in a thinning social network and further loneliness. Similarly, caregivers of people living with chronic conditions in Diefenbeck et al.’s study described feeling lonely and isolated [24].

Most of the reviewed studies found that, by using internet-based platforms, caregivers received social support and accessed resources, all while feeling integrated into a meaningful online social network. Additionally, internet-based platforms helped caregivers gain a sense of social inclusion and belonging as members of an online community where others could understand what they were experiencing. Sullivan described how study participants, knowing that others are going through similar experiences, felt less isolated and more a part of a community [37]. This finding is consistent with the findings of other reviewed studies. For instance, McKechnie and colleagues examined postings on an online forum for carers of people living with dementia and reported that the notion of “social similarity” was especially beneficial to them [33]. This notion refers to finding others who are in the same situation. Caregivers gained comfort in knowing that they were not alone when they connected with others who could understand the challenges they faced and what they were experiencing. Kim’s participants described the sense of belonging that they got from being Facebook group members as particularly effective in reducing their feelings of isolation [29]. These participants explained that this sense of belonging they gained resulted from the personal connections they made in the group and the camaraderie that developed among caregivers who had gone through similar experiences [29]. Furthermore, when Stephen et al. examined an online cancer support group, they found that carers valued the closeness they felt with other group members [35]. Participants’ experiences resonated with others, and there was a common understanding among group members. This understanding forged strong bonds among group members, producing a sense of belonging that, in turn, reduced loneliness. Not only did group members feel understood, they felt accepted.

Dam and colleagues found that an online social-support intervention called *Inlife* benefitted caregivers of people living with dementia [22]. The authors used the following measures to evaluate the intervention’s outcomes: Multidimensional Scale of Perceived Social Support (MSPSS), Social Support List (SSL-12), and Loneliness Scale (LS). The evaluation showed improvement in perceived family support and reduced loneliness in the highly active *Inlife* user group. Users also reported that *Inlife* helped them share their experiences with others. Similarly, Marziali and Garcia used the MSPSS and content analysis to examine how caregivers experienced two interventions: online messaging and online video conferencing [32]. Their MSPSS results demonstrated no significant change in perceived social support before and after the online messaging group. However, for the online video-conferencing group, the MSPSS demonstrated significant positive changes in perceived social support. The authors’ qualitative analysis of follow-up interviews with participants showed mixed reactions to the online-messaging intervention. In contrast, participants in the video-conferencing group felt very connected through the sharing of experiences by multiple caregivers. They described receiving a lot of emotional and informational support from other caregivers.

After studying an online support group for caregivers of people with Alzheimer’s, Kruk found that exposure to other carers’ parallel experiences as well as recognition and validation of their own experiences reduced participants’ feeling of loneliness and social isolation [30]. Blusi et al. also reported that caregivers appreciated the ability to visit other caregivers through the internet [21]. These visits allowed them to leave their homes at least virtually and thus reduced their feelings of isolation. The authors contended that internet-based caregiver support enabled their study participants to form social networks. In addition, Blusi and colleagues highlighted that, for family carers, the internet enabled them to recapture a valued position in society and “be somebody.” [20] The authors argued that internet-based support is particularly important for caregivers in rural areas, because it can reduce loneliness by providing new opportunities for social inclusion.

#### Benefits and challenges of internet-based support

The studies we reviewed reported the various benefits of internet-based platforms that encouraged informal caregivers to find social support on line. Researchers highlighted advantages such as anonymity, instant access, receiving support within one’s own home, and being able to carefully craft messages before posting to online forums [23]. They also indicated that in-person support groups may be challenging to access due to constraints associated with caregiving itself or caregivers’ locations, particularly if they live in rural areas [23]. This was consistent with the findings of Bateman and colleagues, as caregivers in their study reported insufficient access to in-person support [19]. The participants experienced difficulty in leaving their homes due to their caregiving role that led to less access to social or emotional support from friends and family. As a result, their network of immediately available supporters shrank drastically and, consequently, participants resorted to seeking support online.

McKechnie and colleagues added benefits to caregivers that are unique to internet-based support: the honesty enabled by anonymity, flexibility, and continuity of access [33]. Similarly, Darcy and colleagues argued that online forums enable the intimate self-disclosure needed to form online friendships [23]. They also proposed that sharing experiences in online forums can provide hope to others and increase shared identity. In the same vein, Barbabella et al. reported that caregivers found their internet-based intervention, which comprised a web platform that provided information resources and interactive services areas to informal caregivers, to be a safe environment in which to share personal experiences [18]. Caregivers in Anderson et al.’s study also asserted that the social support they received was not bound by geography; they were able to connect with other caregivers regardless of their location and offer support through blogs, an internet-based digital tool [17]. Ultimately, the reviewed articles found that current support resources have significant limitations because they are often available only in person. Researchers suggest that interventions to support informal caregivers through internet-based methods are effective in addressing those limitations by offering flexibility and access to support in caregivers’ homes. However, four studies reported certain technical challenges that existed in internet-based interventions for caregivers [18, 20, 32, 35]. For instance, Barbabella and colleagues found that some participants mentioned technical or usability issues as a reason for limiting their use of some of the available interactive services [18]. Similarly, some family caregivers in Blusi and colleagues’ study experienced technical problems in the beginning but was able to overcome those problems in a quick manner by obtaining technical support from nurses [20]. The researchers highlighted that technical support and follow ups were important to ensure the success of the internet-based intervention [20]. Furthermore, in their internet-based intervention, Marziali and Garcia also indicated that some caregivers experienced problems with accessing the video conferencing software, which was resolved with the help of technicians. Lastly, some participants in Stephen et al.’s study described sometimes experiencing “technology glitches” when accessing an online support program. While these researchers found that participants experienced technical challenges, they were often resolved with the support of technicians and healthcare professionals in a timely manner.

## Discussion

As the internet becomes more accessible, internet-based platforms are becoming an increasingly common source of health-related communication and a means for informal caregivers to exchange support [40, 41].

Findings from the reviewed studies indicated that caregivers may not have people within their existing social networks who have experience providing care for loved ones, and they often desire to connect with peers who have done so [18, 25, 29, 34, 37]. Caregivers often turn to internet-based platforms that offer anonymity, flexibility, and 24-h access from the comfort of their homes [24, 25, 34, 35]. These benefits are not offered by in-person support programs. By engaging with peers in an online community, participants in the reviewed studies reported regaining a sense of social inclusion and belonging. This is particularly important, given that informal caregivers often face numerous constraints due to their role, resulting in feelings of loneliness and social isolation [42–44]. From our review, it appears that social media platforms have a potential to address caregivers’ needs in complement to other services.

### Limitations of scoping review and the studies reviewed

Given that one of the inclusion criteria for this scoping review was that studies be in English, the knowledge from studies published in other languages was not captured. Another limitation is that full text studies available through the Ryerson University library database were only included due to the limited budget constraints of the scoping review. Further, the quality of the studies was not analyzed as Arksey and O’Malley’s [14] methodology was used to guide the scoping review. Additionally, it is important to note that the purpose of scoping reviews is to identify and map the available evidence in terms of the volume, nature, and characteristics of the primary research when a topic has not yet been extensively reviewed [14]. Thus, scoping reviews aim to provide a descriptive overview of the reviewed articles without critically appraising the individual studies [14].

When we compared studies, all outcome measures and reported outcomes were weighted equally, even though some studies may have used more reliable outcome measures or provided more comprehensive assessments of social support and inclusion.

No reviewed study reported recruiting people under the age of 18; therefore, the findings of our scoping review may not be transferable to young informal caregivers. Almost half of all the studies did not report participants’ demographic characteristics (e.g., age, ethnicity, and gender), which made it difficult for us to conclude to what extent different populations are being served within internet-based support interventions. Many studies evaluated interventions that were broadly defined or provided few details about the intervention they evaluated, making it difficult to compare interventions between studies.

### Areas for future research

In this review, there was an over-representation of studies from the United States and Europe. Accordingly, studies on caregivers’ use of online support in regions with different ethno-cultural and socio-economic contexts, such as the Middle East and Asia, were missing from the review. Future research is needed to evaluate the impact and effectiveness of digital tools targeting caregivers under the age of 18 and among populations diverse in racial and ethnic background. Considering that an overwhelming number of the reviewed studies recruited mostly women, how male caregivers use online tools needs to be captured. Future research could also investigate the needs for support and the use of online tools among informal caregivers with non-binary gender identities.

To enable future reviewers to compare the efficacy and impact of different interventions, it would be helpful if future evaluations could report the interventions’ components more systematically and in more detail. For example, authors can describe various components of their interventions, such as duration, content, process, and development. In our review, many studies were excluded because they evaluated only usability and acceptability of the technology in general and not specifically social support (including experiences of social isolation or social needs of the informal caregivers). Evaluation of interventions should also include efficacy and impact.

## Conclusion

Research has shown that the health of informal caregivers is a major influence on care recipients’ health and well-being, as informal caregivers’ declining health can negatively impact care recipients’ health [45]. Moreover, if informal caregivers lack effective coping styles, this can lead to poorer health outcomes for care recipients, such as increased risk for falls and development of preventable secondary complications (i.e., pressure sores) [46]. It is evident from the reviewed studies that informal caregivers often experience poor health outcomes as a result of their caregiving role, such as loneliness, increased stress, and limited social support. The findings from our scoping review suggest that interventions using existing internet-based digital tools, such as social media platforms, can be a cost-effective and convenient strategy for developing support programs to help informal caregivers form online communities, gain support from each other, and access resources. Promoting well-being among informal caregivers through internet-based interventions can also yield more positive health outcomes for their care recipients.

More importantly, health and social care professionals, such as those in community agencies, doctors, nurses, and social workers, can also leverage digital tools to provide informal caregivers with informational support and resources. Professionals can use the themes identified in this scoping review to engage with and promote the health of care recipients and their caregivers. By considering the ways in which caregivers use internet-based digital tools for information and support, health and social care professionals can better understand their needs. Finally, increased funding and mandates to develop and market high-quality, online social support for informal caregivers could significantly reduce their social isolation.

## Supplementary information

**Additional file 1.** Literature Search Strategies

## Data Availability

Not applicable

## Abbreviations

LS: Loneliness Scale
MSPSS: Multidimensional Scale of Perceived Social Support
SSL-12: Social Support List
