# Supplementary material for: The role of internet-based digital tools in reducing social isolation and addressing support needs among informal caregivers: a scoping review
Source: BMC Public Health. 2019 Nov 9;19:1495. doi: 10.1186/s12889-019-7837-3 (PMC6842183; doi:10.1186/s12889-019-7837-3)
Supplement: Supplementary file 1 — Additional file 1. Literature Search Strategies [file 12889_2019_7837_MOESM1_ESM.docx]

**Search Strategy for Peer-reviewed Literature in Academic Databases**

| **Database** | **Hits** | **Unique Hits**  **(Duplicates Removed)** |
| --- | --- | --- |
| Medline-OVID | 1171 | 947 |
| EMBASE-OVID | 170 | 88 |
| PsycINFO-OVID | 791 | 343 |
| CINAHL-EBSCO | 849 | 340 |
| **Total** | 2977 | 1718 |

**Search Strategy for Medline Databases: OVID Medline Epub Ahead of Print, In-Process & Other Non-Indexed Citations, Ovid MEDLINE(R) Daily, and Ovid MEDLINE(R)**

Date range: 1946 to Present

| **Search**  **#** | **Search Terms** | **Results** |
| --- | --- | --- |
| 1 | internet/ or blogging/ or social media/ | 71,950 |
| 2 | digital/ or social media/ or online/ or internet/ or web/ or blogs/ or website | 31,3576 |
| 3 | 1 or 2 | 33,6853 |
| 4 | caregivers | 32,027 |
| 5 | family or families or parent? or friend? or relative? or spouse? or partner? or husband? or wife or wives or child or children or close person? or significant other? or informal or unpaid | 2,937,194 |
| 6 | 4 and 5 | 21,323 |
| 7 | ((family or families or parent? or friend? or relative? or spouse? or partner? or husband? or wife or wives or child or children or close person? or significant other? or informal or unpaid) adj5 (carer* or caregiv* or (care adj2 giv*))) | 26,845 |
| 8 | 6 or 7 | 35,437 |
| 9 | 3 and 8 | 1365 |
| 10 | limit 9 to English language | 1323 |
| 11 | remove duplicates from 10 | 1171 |

**Search Strategy for EMBASE**

Date range: 1974 to December 08, 2017

| **Search**  **#** | **Search Terms** | **Results** |
| --- | --- | --- |
| 1 | blogging/ or e-mail/ or internet/ or social media/ or text messaging/ or videoconferencing/ or webcast | 119,583 |
| 2 | digital or social media or online or internet or web or blogs or website | 374,374 |
| 3 | 1 or 2 | 422,725 |
| 4 | caregiver | 61,199 |
| 5 | caregiver support | 2507 |
| 6 | 4 or 5 | 63,069 |
| 7 | family or families or parent? or friend? or relative? or spouse? or partner? or husband? or wife or wives or child or children or close person? or significant other? or informal or unpaid | 3,329,857 |
| 8 | 6 and 7 | 37,170 |
| 9 | ((family or families or parent? or friend? or relative? or spouse? or partner? or husband? or wife or wives or child or children or close person? or significant other? or informal or unpaid) adj5 (carer* or caregiv* or (care adj2 giv*))) | 32,081 |
| 10 | 8 or 9 | 48,091 |
| 11 | 3 and 10 | 2497 |
| 12 | limit 11 to exclude Medline journals | 196 |
| 13 | limit 12 to English language | 182 |
| 14 | remove duplicates from 13 | 170 |

**Search Strategy for PsycINFO**

Date range: 1987 to 2^nd^ week of November, 2017

| **Search**  **#** | **Search Terms** | **Results** |
| --- | --- | --- |
| 1 | caregivers | 24,521 |
| 2 | family or families or parent? or friend? or relative? or spouse? or partner? or husband? or wife or wives or child or children or close person? or significant other? or informal or unpaid | 853,564 |
| 3 | 1 and 2 | 18,702 |
| 4 | ((family or families or parent? or friend? or relative? or spouse? or partner? or husband? or wife or wives or child or children or close person? or significant other? or informal or unpaid) adj5 (carer* or caregiv* or (care adj2 giv*))) | 23,872 |
| 5 | 3 or 4 | 29,588 |
| 6 | internet/ or blog/ or electronic learning/ or exp social media/ or exp websites/ | 38,157 |
| 7 | digital or social media or online or internet or web or blogs or website* | 122,494 |
| 8 | 6 or 7 | 125,721 |
| 9 | 5 and 8 | 827 |
| 10 | limit 9 to English language | 791 |
| 11 | remove duplicates from 10 | 791 |

**Search Strategy for CINAHL-EBSCO**

| \| **Search**  **#** \| **Query** \| **Search Limiters/Expanders** \| **Results** \| \| --- \| --- \| --- \| --- \| \| S13 \| S5 AND S11 \| Limiters - English Language; Exclude Medline records  Search modes - Boolean/Phrase \| 849 \| \| S12 \| S5 AND S11 \| Search modes - Boolean/Phrase \| 1,529 \| \| S11 \| S6 OR S7 OR S8 OR S9 OR S10 \| Search modes - Boolean/Phrase \| 190,423 \| \| S10 \| TX (digital or social media or online or internet or web or blogs or website*) \| Search modes - Boolean/Phrase \| 190,423 \| \| S9 \| (MH “Digital Divide”) \| Search modes - Boolean/Phrase \| 197 \| \| S8 \| (MH “Blogs”) \| Search modes - Boolean/Phrase \| 2,102 \| \| S7 \| (MH “Social Media”) \| Search modes - Boolean/Phrase \| 4,680 \| \| S6 \| (MH “Internet”) \| Search modes - Boolean/Phrase \| 28,580 \| \| S5 \| S3 OR S4 \| Search modes - Boolean/Phrase \| 23,402 \| \| S4 \| TX ((family or families or parent? or friend? or relative? or spouse? or partner? or husband? or wife or wives or child or children or close person? or significant other? or informal or unpaid) N5 (carer* or caregiv* or (care N2 giv*))) \| Search modes - Boolean/Phrase \| 16,280 \| \| S3 \| ((family or families or parent? or friend? or relative? or spouse? or partner? or husband? or wife or wives or child or children or close person? or significant other? or informal or unpaid)) AND (S1 OR S2) \| Search modes - Boolean/Phrase \| 15,093 \| \| S2 \| (MH “Caregiver Support”) \| Search modes - Boolean/Phrase \| 3,535 \| \| S1 \| (MH “Caregivers”) \| Search modes - Boolean/Phrase \| 20,815 \|   **Grey Literature Searched**  <https://www.canada.ca/en/health-canada.html> Health Canada  <https://www.nih.gov/> National Institutes of Health  <https://www.healthit.gov/> Health Information Technology  <http://www.pewresearch.org/>  Pew Research Center  [http://www.caregiving.org](http://www.caregiving.org/)  National Alliance for Caregiving  <https://thecaregivernetwork.ca/> The Caregiver Network  <http://www.familycaregiversbc.ca/> Family Caregivers of British Columbia  <https://www.caregiversalberta.ca/> Caregivers Alberta  <http://caregiversns.org/> Caregivers Nova Scotia  <https://www.fraserinstitute.org/> The Fraser Institute  <http://www.changefoundation.ca/> The Change Foundation  <https://www.caregiver.org/> Family Caregiver Alliance  <http://www.caregiverexchange.ca/> Caregiver Exchange  <http://alz.to/get-help/caregiver-framework/>  The Caregiver Project for Seniors  <http://www.centralregistry.ca/crp/home>  Caregiver ReCharge Services  [http://ontario.cmha.ca](http://ontario.cmha.ca/)  Canadian Mental Health Association - Ontario  <https://youngcarersproject.wordpress.com/> Young Carers Project  <https://uwaterloo.ca/murray-alzheimer-research-and-education-program/> Murry Alzheimer Research and Education Program  <http://www.ocsa.on.ca/>  Ontario Community Support Association  <https://ofcan.org/>  Ontario Family Caregivers Advisory Network  <https://caregivingmatters.ca/> Caregiving Matters Caregiving Matters  <http://www.cfhi-fcass.ca/Home.aspx>  Canadian Foundation for Healthcare Improvement  [https://www.nextstepincare.org](https://www.nextstepincare.org/) Next Step in Care  <http://thecaregiverslighthouse.com/> The Caregiver’s Lighthouse  <http://www.caregiveraction.org/>  Caregiver Action Network  <http://www.familycaregiversvoice.ca/> Family Caregivers Voice  <https://www.caregiverstress.com/> Home Instead Senior Care  <http://thefamilycaregiver.com/> The Family Caregiver  <http://www.von.ca/en/>  Victorian Order of Nurses  [https://cancerchat.desouzainstitute.com](https://cancerchat.desouzainstitute.com/) The DeSouza Institute  [http://mehi.masstech.org](http://mehi.masstech.org/)  Massachusetts eHealth Initiative  [http://www.carp.ca](http://www.carp.ca/)  Canadian Association or Retired People  <http://www.canadacares.org/> Canada Cares  <https://www.caphc.org/aboutcfan/>  Canadian Family Advisory Network  <http://www.cdnhomecare.ca/> Canadian Homecare Association  [http://www.carerscanada.ca](http://www.carerscanada.ca/) Carers Canada  <http://www.ontariocaregivercoalition.ca/> Ontario Caregivers Coalition  <http://www.internationalcarers.org/>  International Alliance of Carer Organizations  [https://www.carersuk.org](https://www.carersuk.org/) Carers UK  <https://familycarers.ie/>  Family Carers Ireland  <https://www.aarp.org/> American Association of Retired Persons  [http://www.eurocarers.org](http://www.eurocarers.org/) Eurocarers |
| --- | --- | --- | --- | --- | --- | --- | --- | --- | --- | --- | --- | --- | --- | --- | --- | --- | --- | --- | --- | --- | --- | --- | --- | --- | --- | --- | --- | --- | --- | --- | --- | --- | --- | --- | --- | --- | --- | --- | --- | --- | --- | --- | --- | --- | --- | --- | --- | --- | --- | --- | --- | --- | --- | --- | --- | --- |
